# The 5′ Untranslated Region of Human Bocavirus Capsid Transcripts Regulates Viral mRNA Biogenesis and Alternative Translation

**DOI:** 10.1128/JVI.00443-18

**Published:** 2018-10-12

**Authors:** Xiaoqian Liu, Sujuan Hao, Zhen Chen, Huanzhou Xu, Hanzhong Wang, Min Huang, Wuxiang Guan

**Affiliations:** a Center for Emerging Infectious Diseases, Wuhan Institute of Virology, Chinese Academy of Sciences, Wuhan, Hubei, China; b University of Chinese Academy of Sciences, Beijing, China; c Department of Laboratory Medicine, Tongji Hospital, Tongji Medical College, Huazhong University of Science and Technology, Wuhan, China; University of Utah

**Keywords:** HBoV1, alternative translation, uATG, 5′ UTR

## Abstract

The capsid mRNA transcripts of human bocavirus 1 (HBoV1) can be generated by alternative splicing from the mRNA precursor transcribed from the P5 promoter. However, the alternative translation regulation mechanism of capsid mRNA transcripts is largely unknown. Here we report that the polycistronic capsid mRNA transcripts encode VP1, VP2, and VP3 *in vitro* and *in vivo*. The 5′ untranslated regions (UTRs) of capsid mRNA transcripts, which consist of exons, affected not only the abundance of mRNA but also the translation pattern of capsid proteins. Further study showed that exons 2 and 3 were critical for the abundance of mRNA, while exon 4 regulated capsid translation. Alternative translation of capsid mRNA involved a leaky scan mechanism. Mutating the upstream ATGs (uATGs) located in exon 4 resulted in more mRNA transcripts polyadenylated at the proximal polyadenylation [(pA)p] site, leading to increased capsid mRNA transcripts. Moreover, uATG mutations induced more VP1 expression, while VP3 expression was decreased, which resulted in less progeny virus production. Our data show that the 5′ UTR of HBoV1 plays a critical role in the modulation of mRNA abundance, alternative RNA processing, alternative translation, and progeny virus production.

**IMPORTANCE** Alternative translation of HBoV1 capsid mRNAs is vital for the viral life cycle, as capsid proteins perform essential functions in genome packaging, assembly, and antigenicity. The 5′ untranslated regions (UTRs) of capsid mRNAs are generated by alternative splicing, and they contain different exons. Our study shows that the 5′ UTR not only modulates mRNA abundance but also regulates capsid expression. Two upstream ATGs (uATGs) that were upstream of the capsid translation initiation site in the 5′ UTR were found to affect viral capsid mRNA polyadenylation, alternative translation, and progeny virus production. The results reveal that uATGs play an important role in the viral life cycle and represent a new layer to regulate HBoV1 RNA processing, which could be a target for gene therapy.

## Abstract (precis)

Alternative translation of HBoV1 capsid mRNAs is vital for the viral life cycle, as capsid proteins perform essential functions in genome packaging, assembly, and antigenicity. The 5′ untranslated regions (UTRs) of capsid mRNAs are generated by alternative splicing, and they contain different exons. Our study shows that the 5′ UTR not only modulates mRNA abundance but also regulates capsid expression. Two upstream ATGs (uATGs) that were upstream of the capsid translation initiation site in the 5′ UTR were found to affect viral capsid mRNA polyadenylation, alternative translation, and progeny virus production. The results reveal that uATGs play an important role in the viral life cycle and represent a new layer to regulate HBoV1 RNA processing, which could be a target for gene therapy.

## INTRODUCTION

Human bocavirus (HBoV) was first identified in 2005 (1). It is the second human pathogen of the genus *Parvovirus* to be identified, with the first being parvovirus B19 (2, 3). Four HBoV variants are commonly detected in respiratory samples or feces from infants (4–6). HBoV1 is prevalent in samples from patients with acute respiratory infections and often coinfects with other respiratory viruses (7–9). HBoV1 infection leads to various clinical manifestations, such as pneumonia (10–13), bronchiolitis (14, 15), and acute otitis media (16, 17), which can be life-threatening.

The genome of HBoV1 is a single-stranded DNA about 5.5 kb in length with terminal hairpins at both ends (18–21). The precursor mRNA transcript (pre-mRNA) is transcribed by the promoter (P5) on the left and is alternatively processed to generate mature mRNA transcripts that encode the viral proteins (22–24). The mRNA transcripts of nonstructural proteins NS1, NS2, NS3, and NS4 are transcribed from the left half of the viral genome (23). The middle of the viral genome contains a small open reading frame (ORF) encoding the NP1 protein, a 22-kDa nuclear phosphoprotein that facilitates viral DNA replication, RNA processing, and the production of capsid mRNAs (24, 25).

The capsid mRNA transcripts are alternatively spliced from pre-mRNA, and the 5′ untranslated regions (UTRs) consist of different combinations of exons (22, 23). The capsid proteins VP1, VP2, and VP3 share a C terminus, and VP2 is translated from a noncanonical initiation site, GUG (24). The N terminus of VP1 contains a phospholipase A2 (PLA2) domain that is essential for parvovirus infectivity (26). NP1 plays a critical role in the expression of the capsid proteins (24). Polyadenylation at the distal polyadenylation [(pA)d or (pA)d2] site also modulates the expression of capsid proteins (27). However, the detailed mechanism that regulates the alternative translation of capsid proteins is still not understood.

Alternative translation from the same mRNA transcript is important for the regulation of protein expression. The majority of eukaryotic cellular mRNAs initiate translation by cap-dependent translation initiation, which depends on the ribosomal recognition of the m^7^G cap (28, 29). In some cases, the small ribosomal subunit can also be recruited to the mRNA by the internal ribosomal entry site (IRES) when cellular conditions inhibit cap-dependent translation initiation (30, 31). The IRES motif has been found in several viral mRNAs, such as hepatitis C virus (HCV) mRNAs (32) and retrovirus mRNAs (33–36), as well as in the mRNAs of RNA viruses that infect invertebrates (37, 38), plants (39, 40), or protozoa (41). For mRNAs that lack a cap structure or an IRES motif, translational regulation can be mediated via other *cis*-acting elements in the 5′ UTR or 3′ UTR (42–44).

In the present study, we found that the tricistronic capsid mRNA transcript encodes the VP1, VP2, and VP3 proteins both *in vivo* and *in vitro*. The 5′ UTRs of the capsid mRNAs regulated not only the abundance of RNA transcripts but also the expression of capsid proteins. The upstream ATGs (uATGs) in exon 4 of the 5′ UTR modulated capsid expression via a leaky scan mechanism and affected progeny virus production. Mutated uATGs in exon 4 before the capsid translation start site altered the viral RNA abundance as well as mRNA processing, indicating that the 5′ UTR plays an important role in the viral life cycle.

## RESULTS

### The 5′ UTR of HBoV1 capsid mRNA transcripts regulated RNA abundance and protein expression.

**FIG 1:**
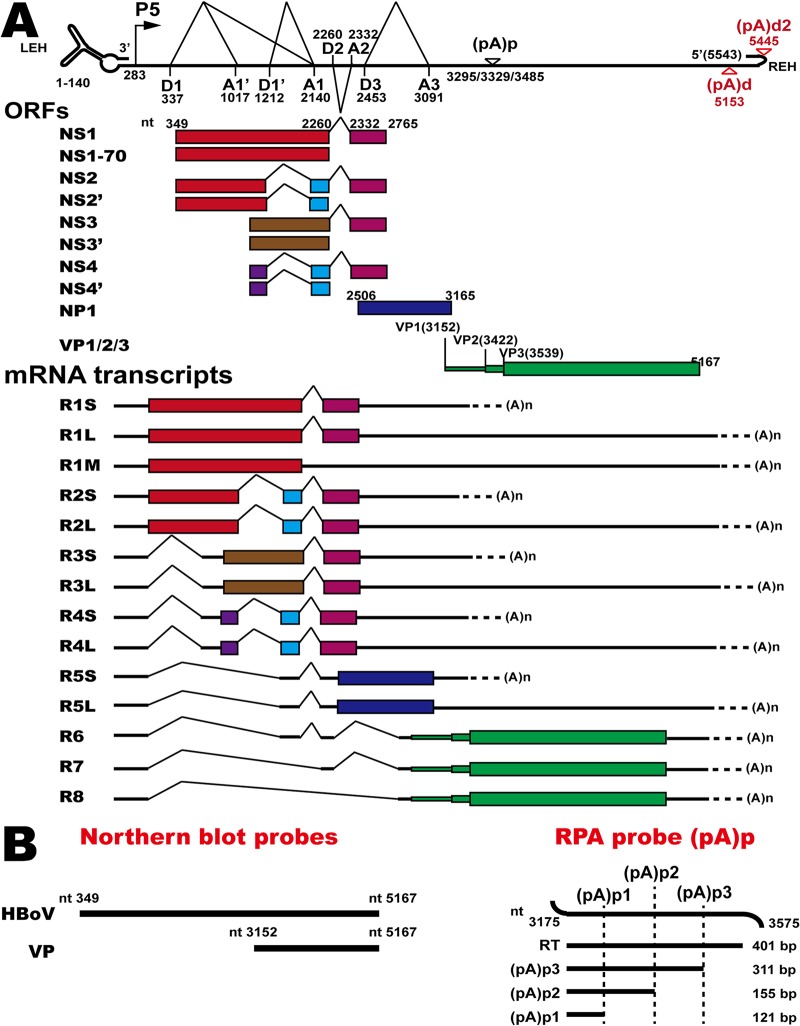
HBoV1 transcription map and probes. (A) Genome structure of HBoV1, the main ORFs, and the major species of viral mRNA transcripts that are alternatively processed. P5, P5 promoter; D, 5′ splice donor site; A, 3′ splice acceptor site; (pA)p, proximal polyadenylation sites; (pA)d, distal polyadenylation site; LEH, left-end hairpin; REH, right-end hairpin. (B) Probes for Northern blotting or the RNase protection assay (RPA). RT, read-through RNA.

**FIG 2:**
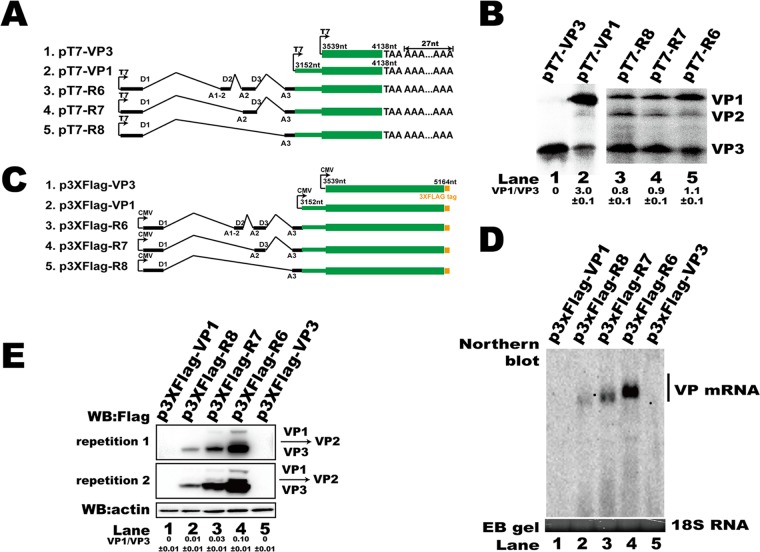
5′ UTR regulated HBoV1 capsid mRNA abundance and protein translation. (A) Diagram of HBoV1 capsid expression constructs with the T7 promoter for the *in vitro* assay. (B) *In vitro* coupled transcription/translation assay. *In vitro* assays were performed according to the manufacturer's instructions. Expressed proteins were run on a 15% SDS-PAGE gel, and the signal was detected with a Cyclone Plus system (PerkinElmer) and analyzed using OptiQuant software. The ratio of VP1 to VP3 is presented at the bottom of the gel. The experiment was repeated at least three times. (C) Diagram of HBoV1 VP cDNA constructs with the cytomegalovirus (CMV) promoter. (D) Northern blot. Ten micrograms of total RNAs prepared from transfected cells were resolved on 1.5% agarose gels, transferred to Hybond-N^+^ membranes, and hybridized with probes spanning nt 349 to 5167. The signal was detected using a ChemiDoc MP imaging system (Bio-Rad). Ethidium bromide (EB)-stained 18S RNA bands are shown as the loading control. (E) Western blot (WB). The lysates of HEK293T cells transfected with the plasmids described in panel C were analyzed using an anti-Flag antibody to detect capsid expression. β-Actin served as the loading control.

HBoV1 capsid protein mRNA transcript R6 (Fig. 1) encodes proteins VP1, VP2, and VP3. However, VP cDNA structures that contain only VP ORFs cannot efficiently generate VP-encoding mRNAs, and VP protein expression is undetectable (24). To investigate the *cis* elements that regulate capsid protein expression, the first 600 nucleotides (nt) of the VP3 ORF and the VP1 unique region (VP1u) sequence with (pT7-R6, pT7-R7, and pT7-R8) or without (pT7-VP1) the 5′ UTR of the R6, R7, and R8 transcripts were inserted into a PCR-BluntII-TOPO vector that contained a 27-nt A tract to increase the *in vitro* translation efficiency (Fig. 2A). *In vitro* coupled transcription/translation with T7 polymerase showed that only truncated VP3 was translated from pT7-VP3 (Fig. 2B, lane 1). However, VP1, VP2, and VP3 were translated from pT7-VP1, indicating that the VP1 mRNA was tricistronic (Fig. 2B, lane 2). Additionally, expression of VP1, VP2, and VP3 was also detected when pT7-R6, pT7-R7, or pT7-R8 was used in an *in vitro* coupled transcription/translation assay (Fig. 2B, lanes 3 to 5). However, the ratio of VP1/VP3 was changed, suggesting that the 5′ UTR participated in regulating the expression of viral capsid proteins.

To further confirm the role of the 5′ UTR in capsid protein expression, p3XFlag-R6, p3XFlag-R7, p3XFlag-R8, p3XFlag-VP1, and p3XFlag-VP3 (Fig. 2C) were transfected into HEK293T cells. The expression of both capsid mRNA and protein was undetected when p3XFlag-VP1 or p3XFlag-VP3 was transfected (Fig. 2D, lanes 1 and 5, and E, lanes 1 and 5). Our result is consistent with that of a previous study that showed that VP ORF cDNA is inefficient for capsid mRNA production (24). However, transfections of p3XFlag-R6, p3XFlag-R7, and p3XFlag-R8 resulted in more mRNA transcripts (Fig. 2D, lanes 2 to 4) and protein expression (Fig. 2E, lanes 2 to 4) as the length of the 5′ UTR increased. The increased VP protein expression was consistent with the increased mRNA level, suggesting that the 5′ UTR plays a role in mRNA production.

### Key *cis* elements in the 5′ UTR of HBoV1 regulated mRNA abundance and protein expression.

**FIG 3:**
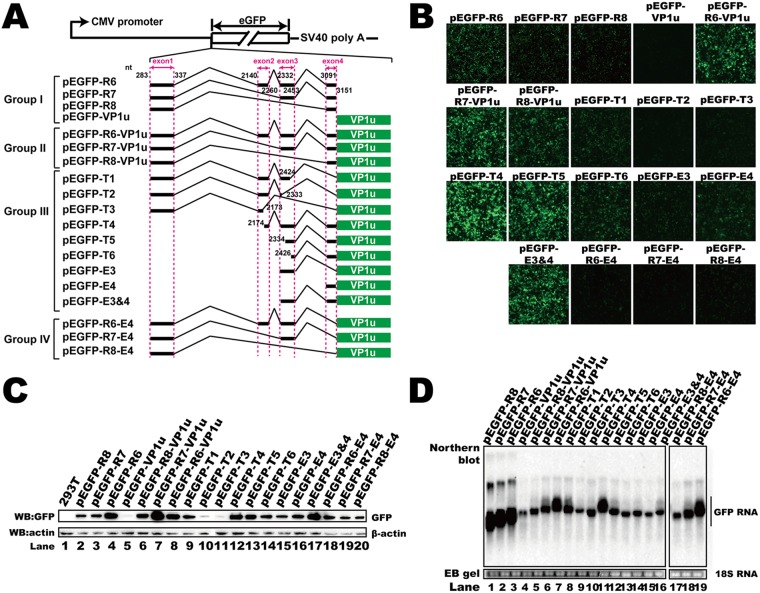
Identification of the *cis* elements in the 5′ UTR that regulated RNA abundance and translation. (A) Diagram of green fluorescent protein (GFP) reporter plasmids. Group I plasmids contained either the entire 5′ UTR or VP1 unique sequences. Group II plasmids contained both the 5′ UTR and VP1u. Group III constructs harbored VP1u with deletions in exons 1 to 4. Exon 4 in the plasmids of group IV was deleted. SV40, simian virus 40. (B) Microscopy of the fluorescent signal when reporter plasmids were transfected. (C) Western blot. The lysates of transfected HEK293T cells were analyzed using anti-GFP antibody, and β-actin served as the loading control. (D) Northern blot. Total RNAs were harvested from transfected cells, and Northern blotting was carried out as described in the legend to Fig. 2D. Ethidium bromide (EB)-stained 18S RNA bands are shown as the loading control. The image in panel D was spliced from two different gels because the samples could not be run in one gel.

To investigate the *cis* elements that regulate HBoV1 mRNA abundance, the 5′ UTR (pEGFP-R6, pEGFP-R7, and pEGFP-R8), VP1u (pEGFP-VP1u), or both of them (pEGFP-R6-VP1u, pEGFP-R7-VP1u, and pEGFP-R8-VP1u) were inserted into pEGFP-N1 (Fig. 3A, groups I and II). Insertion of the 5′ UTRs before the green fluorescent protein (GFP) ORF resulted in more RNA transcripts (Fig. 3D, lanes 1 to 3 and 5 to 7) than insertion of the VP1u sequence only (Fig. 3D, lane 4).

**FIG 4:**
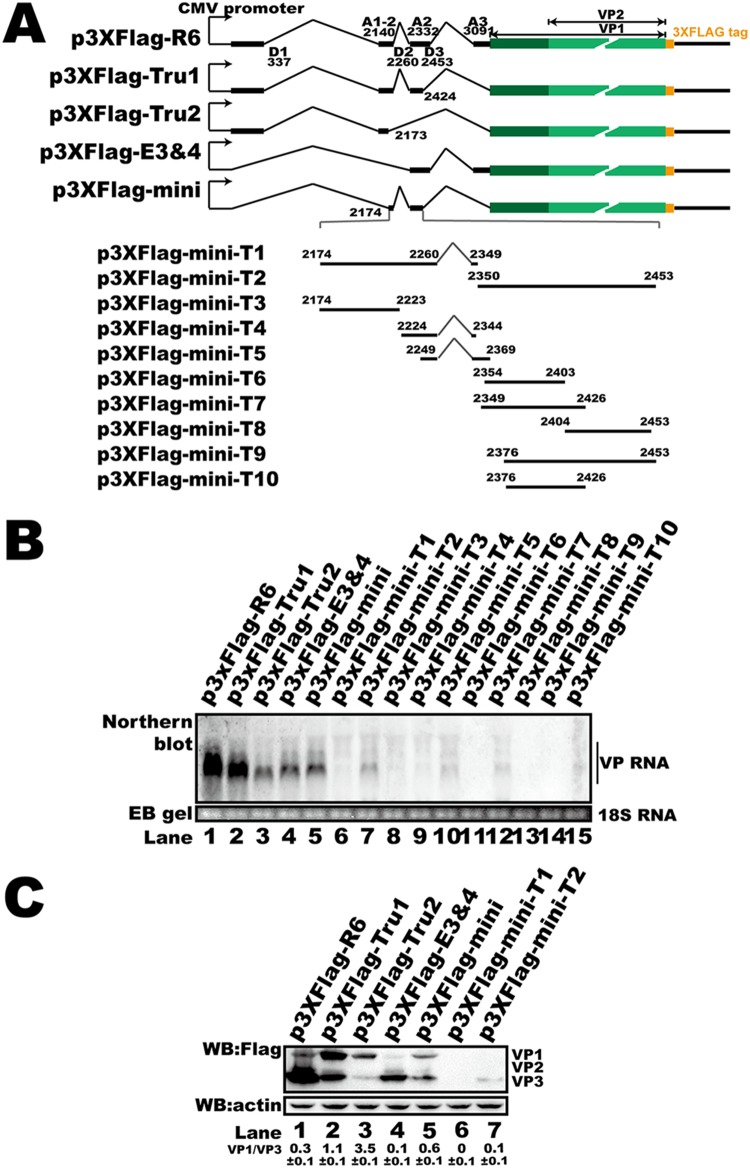
5′ UTR *cis* elements regulated HBoV1 capsid protein expression and mRNA abundance in HEK293T cells. (A) Diagram of capsid expression plasmids. Various lengths of the 5′ UTR sequence from p3XFlag-R6 were deleted to construct p3XFlag-Tru1, p3XFlag-Tru2, p3XFlag-E3 and -E4, and p3XFlag-mini. Plasmids p3XFlag-mini-T1 to p3XFlag-mini-T10 were generated by sequence deletion in exon 2 or exon 3 based on p3XFlag-mini, as indicated. (B) Western blot. The transfected HEK293T cell lysates were analyzed using anti-Flag antibody, and β-actin served as the loading control. (C) Northern blot. Total RNAs were harvested from transfected cells, and Northern blotting was carried out as described in the legend to Fig. 2D. Ethidium bromide (EB)-stained 18S RNA bands are shown as the loading control.

We next made mutant plasmids based on pEGFP-R6-VP1u (Fig. 3A, groups III and IV) to determine the key *cis* elements that regulate mRNA abundance. The 5′ UTR of these plasmids consisted of different combinations of exons. Interestingly, deletion of exon 4 (Fig. 3A, group IV) led to an unchanged GFP mRNA abundance (Fig. 3D, lanes 5 to 7 versus lanes 17 to 19). Deletion of sequences in exon 2 or 3 resulted in decreased GFP mRNA (Fig. 3D, lanes 8 to 10 and 12 to 16 versus lane 7), indicating that the sequences in these two exons are essential for mRNA abundance. The RNA abundance associated with plasmids containing part of exon 2 (nt 2174 to 2260) and exon 3 was higher than the RNA abundances associated with other truncated plasmids (Fig. 3D, lane 11), supporting the view that exons 2 and 3 participate in regulating RNA abundance. To further confirm our results, exons on p3XFlag-R6, which contained natural sequences of the R6 transcript cDNA, were truncated and the plasmids were transfected into HEK293T cells (Fig. 4A). We found that deletion of exon 2 or 3 resulted in decreased mRNA (Fig. 4C, lanes 3 and 4), which was consistent with our reporter system. The results presented above suggest that exons 2 and 3 regulate RNA abundance.

We next checked the protein expression of the reporter plasmids. Insertion of the VP1u sequence without 5′ UTR sequences resulted in decreased GFP expression compared to the GFP expression associated with plasmids containing HBoV1 5′ UTRs (Fig. 3B and C, lane 5 versus lanes 2 to 4 and 6 to 8). We also found that less GFP was detected either by microscopy or by Western blotting when plasmids that did not contain exon 4 (pEGFP-T1, pEGFP-T2, pEGFP-T3, pEGFP-R6-E4, pEGFP-R7-E4, and pEGFP-R8-E4) were transfected (Fig. 3B and C, lanes 9 to 11, 14, 15, and 18 to 20). Deletion of exon 4 also resulted in more VP1 expression and less VP3 expression (Fig. 4B). These results suggest that there is an important *cis* element in exon 4 that participates in posttranscriptional regulation.

To further look into the minimum *cis* element that is required for capsid RNA biogenesis and protein expression, p3XFlag-mini and its truncated derivatives were transfected into HEK293T cells. We found that only p3XFlag-mini transfection resulted in efficient RNA production and capsid expression (Fig. 4B and C). A weak RNA signal was detected when p3XFlag-mini-T2, p3XFlag-mini-T5, and p3XFlag-mini-T7 were transfected. However, only weak VP3 expression was detected in cells transfected with p3XFlag-mini-T2 (Fig. 4C, lane 7) compared to that detected in cells transfected with the p3XFlag-mini truncated derivatives, such as p3XFlag-mini-T1 (Fig. 4C, lane 6).

Collectively, these results suggest that exons 2 and 3 are required for efficient VP mRNA expression, while exon 4 is essential for VP protein alternative translation.

### The IRES motif did not participate in the regulation of HBoV1 VP protein translation.

**FIG 5:**
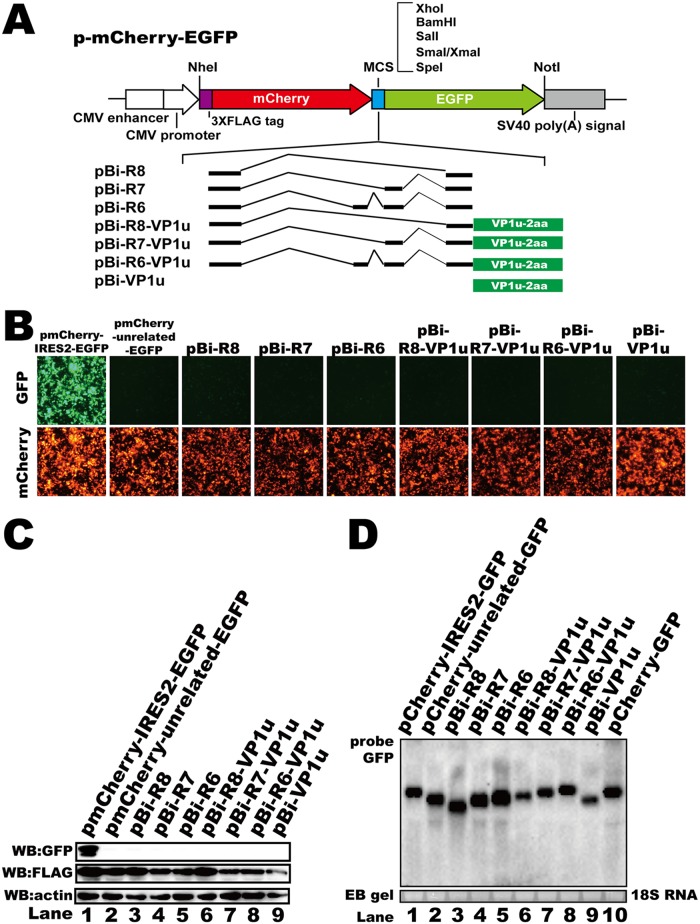
The alternative translation of VP1 and VP2 was IRES independent. (A) Diagram of bicistronic fluorescent reporter constructs. The 3×Flag tag, the simian virus 40 poly(A) signal, and multiple-cloning sites (MCS) are denoted. (B) Microscopy images of fluorescent signals. Images of the green fluorescent protein (GFP) fluorescent signals are on the top, and images of the mCherry fluorescent signals are on the bottom (in images of the same visual fields). (C) Western blot. The lysates of transfected HEK293T cells were analyzed using anti-GFP and anti-Flag antibodies. β-Actin served as the loading control. (D) Northern blot. Total RNAs were harvested from transfected cells, and Northern blotting was carried out as described in the legend to Fig. 2D. Ethidium bromide (EB)-stained 18S RNA bands are shown as the loading control.

IRES elements, which result in alternative translation, have been discovered in many viral mRNAs (45–48). To test whether the alternative translation of HBoV1 VP proteins involved IRES-induced translation initiation, a bicistronic fluorescent reporter plasmid was constructed (Fig. 5A). In this reporter system, mCherry was translated by a cap-dependent mechanism, while enhanced green fluorescent protein (eGFP) was translated only if the preceding sequence contained an IRES element. The 5′ UTR with (pBi-R6-VP1u, pBi-R7-VP1u, and pBi-R8-VP1u) or without (pBi-R6, pBi-R7, and pBi-R8) the VP1u sequence was inserted into this vector between the two fluorescent protein ORFs to verify whether any of these sequences allowed expression of both fluorescent proteins (Fig. 5A). The encephalomyocarditis virus (ECMV) IRES was inserted as a positive control (pmCherry-IRES2-EGFP). Both mCherry and eGFP expression was detected by microscopy when pmCherry-IRES2-EGFP was transfected into HEK293T cells (Fig. 5B, pmCherry-IRES2-EGFP). However, no eGFP expression was detected either by microscopy (Fig. 5B) or by Western blotting (Fig. 5C) when the plasmids containing the HBoV1 sequences were transfected, suggesting that no IRES element was involved in HBoV1 capsid protein alternative translation. In addition, the mRNA abundances of these transcripts were enhanced by inserting the 5′ UTR upstream VP1u sequence (Fig. 5D, lane 3 to 8 versus lane 9), which is consistent with the results that showed that capsid mRNAs were upregulated by 5′ UTRs.

### Alternative translation of VP proteins was based on a ribosomal leaky scanning mechanism.

**FIG 6:**
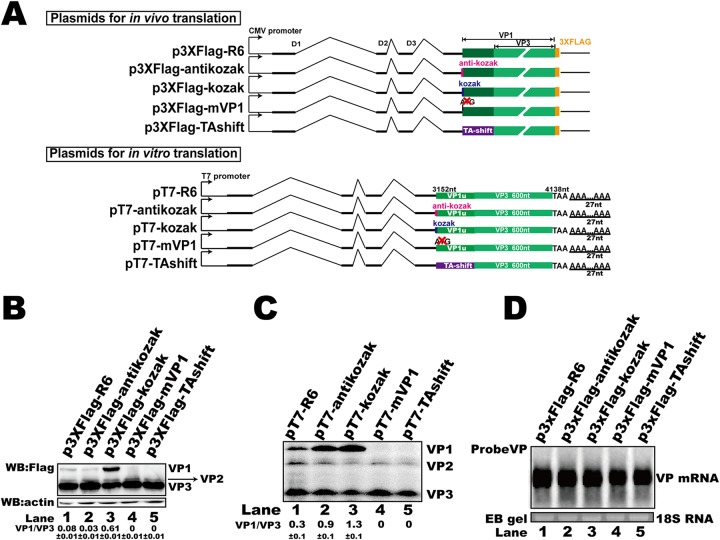
The alternative translation of HBoV1 VP proteins was based on ribosomal leaky scanning. (A) Diagram of plasmids used for *in vitro* or *in vivo* translation. (B) Western blot. The lysates of transfected HEK293T cells were analyzed using anti-Flag antibody, and β-actin served as the loading control. (C) *In vitro* coupled transcription/translation assay. *In vitro* assays were performed as described in the legend to Fig. 2B. (D) Northern blot. Total RNAs were harvested from transfected cells, and Northern blotting was carried out as described in the legend to Fig. 2D. Ethidium bromide (EB)-stained 18S RNA bands are shown as the loading control.

As an IRES element was not involved in the alternative translation of capsid proteins, the potential mechanism by which VP proteins were expressed from the same mRNA transcript probably involved leaky ribosomal scanning with an upstream start codon that is suboptimal (29). To this end, a Kozak sequence (p3XFlag-kozak) or an anti-Kozak sequence (p3XFlag-antikozak) was inserted before the VP1 ORF (Fig. 6A). The transfection of p3XFlag-kozak into HEK293T cells resulted in elevated expression of VP1 but did not affect the RNA abundance (Fig. 6B, lane 3, and D, lane 3). When p3XFlag-antikozak was transfected, VP3 expression increased but VP1 expression was not clearly changed (Fig. 6B, lane 2). The RNA abundance associated with this mutation was not changed compared to that associated with the original expression vector, p3XFlag-R6 (Fig. 6D, lane 2). These results show that the translational context of the VP1 start site affected the expression of VP3, indicating that leaky scanning is involved in the regulation of capsid expression. Mutating the VP1 translation start codon (p3XFlag-mVP1) resulted in a loss of VP1 expression (Fig. 6B, lane 4) but did not affect VP mRNA abundance (Fig. 6D, lane 4) or VP3 expression (Fig. 6B, lane 4). To further confirm our results, a Kozak sequence, an anti-Kozak sequence, a VP1 translation start site knockout mutation, or a TA shift mutation was introduced into the *in vitro* coupled transcription/translation plasmids. The *in vitro* results showed that the Kozak or anti-Kozak sequence altered the efficiency of VP1 expression (Fig. 6C, lanes 2 and 3), while the start site knockout mutation or TA shift mutation abolished the expression of VP1 but did not change VP3 translation (Fig. 6C, lanes 4 and 5), which is consistent with previous results.

Taken together, these results imply that ribosome leaky scanning is involved in the alternative translation regulation of HBoV1 capsid protein expression.

### uATGs in the 5′ UTR mediated alternative translation of capsid proteins.

**FIG 7:**
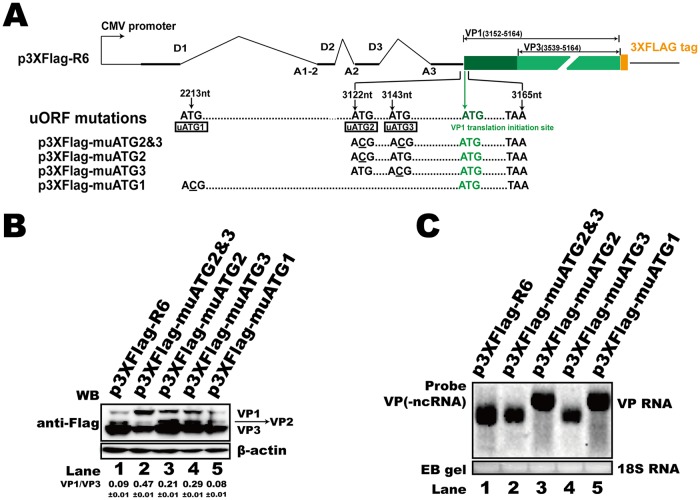
uATGs influenced HBoV1 capsid protein expression. (A) Diagram of HBoV1 uATG-mutated constructs. The positions of uATG1, uATG2, and uATG3 are denoted by their nucleotide sequence numbers. (B) Western blot. The transfected HEK293T cell lysates were analyzed using anti-Flag antibody, and β-actin served as the loading control. (C) Northern blot. Total RNAs were harvested from transfected cells, and Northern blotting was carried out as described in the legend to Fig. 2D. Ethidium bromide (EB)-stained 18S RNA bands are shown as the loading control. ncRNA, noncoding RNA.

The 370-nt 5′ UTR of the HBoV1 R6 transcript contains three uATGs (uATG1, uATG2, and uATG3), which reflect three corresponding upstream ORFs (uORFs) that share the same TAA stop codon with the NP1 ORF (Fig. 7A). A uORF is a common means to regulate the translation of downstream genes. To characterize the function of these uATGs, we replaced ATG with ACG in the capsid expression plasmid p3XFlag-R6, as shown in Fig. 7A. These uATG mutant plasmids were transfected into HEK293T cells, and Western blotting was performed to check the capsid protein expression. The results demonstrated that mutation of the first uATG (uATG1) in exon 2 did not affect capsid protein expression (Fig. 7B, lane 5) and that the RNA abundance was not changed (Fig. 7C, lane 5). However, the uATG2 mutation (p3XFlag-muATG2) or uATG3 mutation (p3XFlag-muATG3) led to the increased expression of VP1 and VP2 translation but had no effect on VP3 expression (Fig. 7B, lanes 3 and 4). When both uATG2 and uATG3 were mutated (p3XFlag-muATG2 and -3), the efficiency of VP2 and VP3 expression decreased, while the expression of VP1 increased (Fig. 7B, lane 2). The RNA abundance was slightly affected (Fig. 7C, lane 2). The results presented above suggest that uATG2 and uATG3 play an essential role in the regulation of capsid protein alternative translation. Interestingly, the mutation of uATG1 or uATG2 induced alternative polyadenylation of the viral capsid RNA (Fig. 7C, lanes 3 and 5).

### uATGs regulated RNA abundance, protein expression, and progeny virus production.

**FIG 8:**
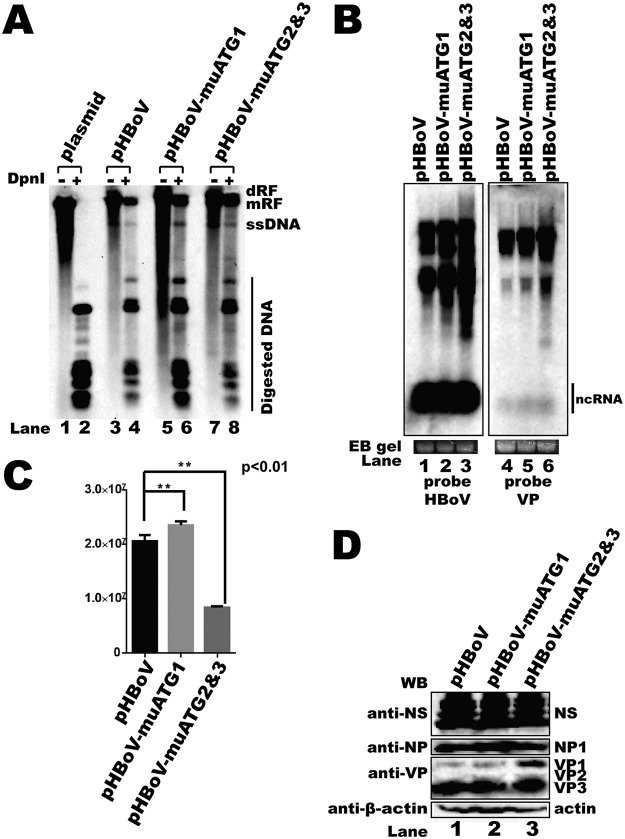
uATG2 and uATG3 knockout mutations in an HBoV1 infectious clone decreased viral progeny production. (A) Southern blot. Hirt DNA was isolated from HEK293T cells transfected with plasmids, as indicated, at 48 h posttransfection. The DpnI-digested fragments were resolved on 1% agarose gels and transferred to a Hybond-N^+^ membrane, followed by hybridization with an HBoV1 probe spanning from nt 1 to 5543. dRF, double replication form; mRF, monomer replication form; ssDNA, single-stranded DNA. (B) Northern blot. Total RNAs were harvested from transfected cells, and Northern blotting was carried out as described in the legend to Fig. 2D. Ethidium bromide (EB)-stained 18S RNA bands are shown as the loading control. The images in panel B were taken from different areas of the same photo or different gels and then joined together. (C) Quantitative PCR (qPCR) for the HBoV1 genome. Cell lysates of uATG-mutated HBoV1 infectious clones were prepared by four cycles of freezing and thawing, followed by DNase I digestion. The progeny viruses were separated by cesium chloride (CsCl) density gradient centrifugation, and genomic DNA was prepared as the template for qPCR. Means and standard deviations were calculated from the results of at least three independent experiments. The pHBoV plasmid was used as a control to establish a standard curve for absolute qPCR (*E* = 105.0%, *R*^2^ = 0.999, slope = −3.208). E, efficiency. (D) Western blot. uATG-mutated infectious clones were transfected into HEK293T cells, and Western blotting was performed with anti-NS, anti-NP, and anti-VP antibodies.

To test the functions of the uATGs in a more natural context, uATG1 (pHBoV-muATG1) or both uATG2 and uATG3 (pHBoV-muATG2 and -3) were mutated in an HBoV1 infectious clone. The mutated plasmids were transfected into HEK293T cells. DNA, RNA, proteins, and progeny virus were harvested 2 days after transfection. Viral genome DNA replication was not affected when uATG1 was mutated (Fig. 8A, lanes 5 and 6). The Western blotting results show that the expression of nonstructural proteins NS and NP1 was not changed (Fig. 8D, lane 2). Interestingly, the genomic copy number of the progeny viruses was increased when uATG1 was mutated (Fig. 8C). Transfection of pHBoV-muATG2 and -3 also resulted in little effect on viral genomic DNA replication (Fig. 8A, lanes 7 and 8) or the expression of nonstructural proteins (Fig. 8D, lane 3). However, VP1 expression was increased when uATG2 and -3 were mutated, which is consistent with our previous result showing that uAUG2 and -3 regulate capsid protein expression. Moreover, progeny virus production was significantly impaired (Fig. 8C), while the overall RNA abundance was increased when an infectious clone with uATG2 and -3 mutations was transfected (Fig. 8B, lane 3).

### uATGs modulated RNA processing.

**FIG 9:**
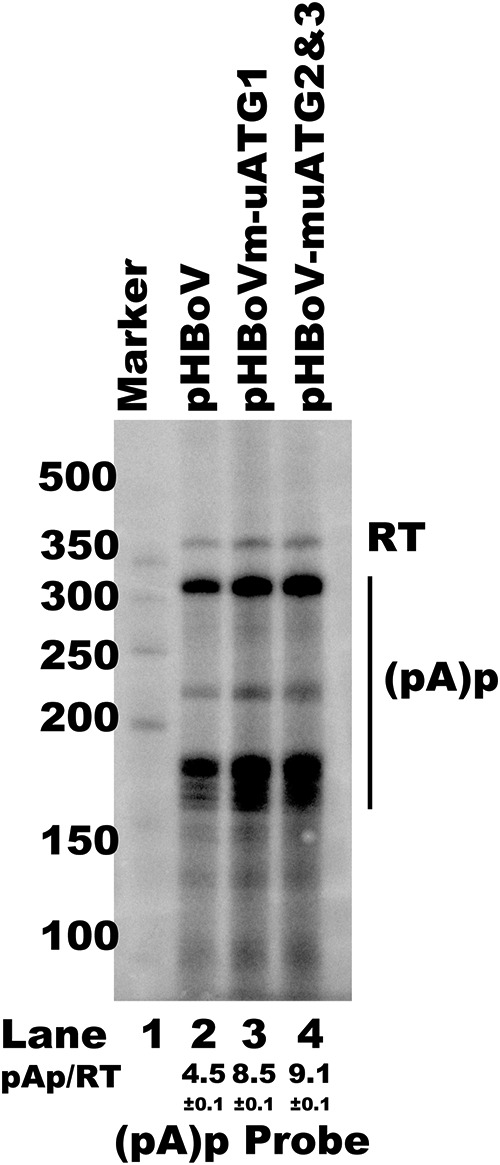
uATG mutations in an HBoV1 infectious clone altered viral RNA processing. RNase protection assay (RPA) with analysis of viral RNA polyadenylation at the (pA)p site. Ten micrograms of total RNAs prepared from HEK293T cells transfected with plasmids, as indicated, was protected by the (pA)p site-specific probe. RT, read through RNAs. The ratios of RNA polyadenylated at the (pA)p site versus read-through RNA are indicated as the mean and standard deviation. The numbers on the left are molecular size markers.

As the uATG2 and -3 mutations led to an amino acid change in NP1, which is involved in RNA processing, we next checked whether uATG mutations affected polyadenylation at the proximal polyadenylation [(pA)p] site. Most RNA was polyadenylated at the (pA)p site when the wild-type infectious clone was transfected into HEK293T cells (Fig. 9, lane 2). To our surprise, mutation of uATG1 or uATG2 and -3 in the infectious clone resulted in more RNA polyadenylation at the (pA)p site (Fig. 9, lane 2 versus lanes 3 and 4).Taken together, these results indicate that the uATGs are essential *cis* elements in the HBoV1 5′ UTR which are required for RNA processing and virus progeny production.

## DISCUSSION

HBoV1 capsid proteins could not be efficiently expressed from VP cDNA (24). In the present study, we found that the 5′ UTR of capsid mRNAs plays essential roles in both capsid mRNA accumulation and alternative translation. Further study showed that key *cis* elements in exons 2 and 3 participate in the 5′ UTR-mediated regulation of RNA abundance. The alternative translation of VP proteins was regulated by a uATG-mediated ribosomal leaky scanning mechanism. The uATGs in exon 4 of the 5′ UTR were required for HBoV1 alternative RNA processing and alternative translation. More importantly, the uATGs were essential for HBoV1 progeny virus production.

Capsid mRNA transcripts are polycistronic and encode VP1, VP2, and VP3. NP1 was reported to be a *trans* element required for VP mRNA production and protein expression (23, 24). NP1 is critical not only to the splicing of VP mRNAs at the A3 splice acceptor site but also to the read-through of internal (pA)p sites by VP mRNAs (24). In our previous study, we showed that alternative polyadenylation at the (pA)d site regulates capsid mRNA abundance and protein expression (27). RNA polyadenylation at the (pA)d2 site increases both the abundance of VP transcripts and the expression of VP proteins. However, the detailed mechanism of capsid mRNA alternative polyadenylation is largely unknown.

The 5′ UTR performs critical regulatory functions in the posttranscriptional RNA process (43, 49). Regulatory elements, such as RNA or protein binding sites, uORFs, uATGs, and RNA secondary structures, impact protein translation directly or indirectly (43, 50). In parvovirus B19, the 5′ UTR has been reported to be important for regulating the correct amounts of capsid proteins produced in the translation process (51). A *cis*-acting element within the NS2 gene of Aleutian mink disease parvovirus was found to be required for efficient capsid protein expression (52). Unlike in parvovirus B19, HBoV1 VP RNAs are polycistronic. The 5′ UTR of the HBoV1 R6 transcript is 370 nt long and consists of four exons (exons 1 to 4). We found that the 5′ UTR is critical for capsid mRNA abundance and protein expression. Further study showed that the second and third exons are essential for mRNA accumulation. Without the 87-bp sequence of exon 2 (nt 2174 to 2260) and exon 3, the fluorescent mRNA abundance decreased significantly (Fig. 3D). Deletion of exon 4 did not affect mRNA abundance in the reporter plasmids but dramatically reduced fluorescent protein expression (Fig. 3C and D), indicating that exon 4 contains *cis* elements for regulating VP protein expression. The minimum *cis* elements required for efficient capsid RNA biogenesis and protein expression consist of part of exon 2 (from nt 2147 to nt 2260) and exon 3. Transfection of p3XFlag-mini-T2 resulted in only VP3 expression. Capsid expression was not detected when p3XFlag-mini-T1 (Fig. 4C, lane 6) or other plasmids (from p3XFlag-mini-T3 to p3XFlag-mini-T10) were transfected (data not shown).

In the cap-dependent translation model, protein translation typically begins with the recruitment of ribosomes to the 5′ cap, followed by migration through the 5′ UTR until the first translation initiation site is encountered (29). If the first initiation site is used inefficiently, some of the 40S ribosomal subunits read through it without recognizing it and may initiate translation at a downstream translation initiation site. We found that neither the 5′ UTR nor the VP1u sequence contained an IRES motif (Fig. 5). However, introducing a Kozak sequence before the ATG of VP1 resulted in increased VP1 expression and decreased VP3 expression (Fig. 6). Mutating the VP1 ATG or a TA shift abolished the expression of VP1 but did not affect VP3 translation. The results presented above show that the context around the first ATG of VP1 is important for the capsid protein expression pattern, indicating that the alternative translation of capsid proteins involves a ribosome leaky scanning mechanism in which both the position and the context of the initiation codon influence translational efficiency.

More than 40% of human genes contain uORFs in their 5′ UTRs (53). uORF-dependent translational control is an important layer used to regulate the level of protein expression (54, 55). Sequence analysis showed that three uATGs are present in the 5′ UTR and that they reflecte three uORFs that share the same stop codon as the NP1 ORF. Mutation of the first uATG (uATG1) either in the reporter system (Fig. 7B) or in the infectious clone (Fig. 8D) did not affect the capsid protein expression pattern. However, the abundance of capsid mRNA transcripts was not changed in the reporter system (Fig. 7C, lane 5), but it was slightly enhanced in the infectious clone (Fig. 8B, lane 5). Mutating uATG2 and uATG3 led to significantly enhanced capsid mRNA expression (Fig. 8B, lane 6) as well as VP1 expression, while VP3 expression was decreased (Fig. 8D, lane 3). These results imply that uATGs play pivotal roles in capsid expression. Mutation of uATG2 and uATG3 in the infectious clone resulted in elevated expression of total viral mRNAs (Fig. 8B, lane 6). However, the expression of NS1 and NP1 proteins was not changed.

Viral uORFs have been reported to regulate viral replication and progeny virus production (56). HBoV1 genome replication was not significantly affected by a uATG1 mutation or by both uATG2 and uATG3 mutations (Fig. 8A). However, progeny virus production was severely impaired by uATG2 and uATG3 mutations (Fig. 8C), which indicates that these two uATGs play important roles in the viral life cycle.

The fact that progeny virus production is affected by uATGs or uORF mutations has been reported in viruses (56). However, little is known about the detailed mechanism. We first checked the RNA processing profile using an RNase protection assay (RPA), as uATG mutations influenced the abundance of capsid mRNA. We found that more RNAs were polyadenylated at (pA)p sites when uATG2 and uATG3 were mutated (Fig. 9B). Our results imply that uATG2 and uATG3 play multiple roles, which include enhancing polyadenylation at the (pA)p site and regulating capsid alternative translation.

In this study, we found that exons 2 and 3 in the 5′ UTR regulate capsid mRNA abundance. However, all the results were obtained from the transfection of HEK293T cells, in which HBoV replicates poorly. The results could be different from those that would be obtained with virus-infected natural target cells. The detailed mechanism explaining how *cis* elements interact with host factors or viral proteins to regulate this process remains unknown and under investigation. The uATGs play essential roles in the viral life cycle and could be a potential target for viral therapy. Developing small molecules or drugs to inhibit the function of uATGs may be an effective way to suppress the replication of HBoV1.

## MATERIALS AND METHODS

### Cell line.

The human embryonic kidney cell line HEK293T (ATCC, CRL-11268) was maintained in Dulbecco's modified Eagle medium (DMEM; Invitrogen) supplemented with 10% fetal bovine serum at 37°C in a humidified incubator with 5% CO_2_.

### Transfection.

Four micrograms of plasmids was transfected by use of the Lipofectamine 2000 reagent (Invitrogen) into HEK293T cells cultured in 60-mm dishes according to the manufacturer's instructions.

### Plasmid construction. (i) VP1/VP3 eukaryotic expression plasmids.

The capsid protein expression plasmids p3XFlag-VP1, p3XFlag-VP3, p3XFlag-R6, p3XFlag-R7, and p3XFlag-R8 were constructed by inserting the sequences of the VP1, VP3, R6, R7, and R8 transcripts, respectively, into the vector p3XFlag-CMV-14 (Sigma-Aldrich) (Fig. 2C). p3XFlag-Tru1, p3XFlag-Tru2, p3XFlag-E3 and -E4, and p3XFlag-mini were made by deletion of various lengths of the 5′ UTR sequence from p3XFlag-R6. Plasmids p3XFlag-mini-T1 to p3XFlag-mini-T10 were constructed by sequence deletion in exon 2 or exon 3, as indicated in Fig. 4A.

### (ii) *In vitro* coupled transcription/translation plasmids.

To improve the *in vitro* coupled transcription/translation efficiency, TAA triplets and a 27-nt A tract were inserted into the vector pCR-BluntII-TOPO-T7 between SpeI and XbaI to construct the vector pCR-BluntII-TOPO-T7-22A. The pT7-VP1 vector was constructed by cloning the VP1 unique region (VP1u) sequence and the first 600 nt of the VP3 ORF (nt 3539 to 4138) into pCR-BluntII-TOPO-T7-22A before the TAA triplets. The pT7-VP3 vector contained the first 600 nt of the VP3 ORF. pT7-R6, pT7-R7, and pT7-R8 were constructed by inserting the 5′ UTR of the R6, R7, and R8 transcripts, respectively, into pT7-VP1, as shown in Fig. 2A.

### (iii) pEGFP-N1-based 5′ UTR truncated plasmids.

pEGFP-R6, pEGFP-R7, pEGFP-R8, and pEGFP-VP1u were made by inserting the 5′ UTRs of the R6, R7, and R8 transcripts and VP1u sequence, respectively, into a eukaryotic expression vector that encodes enhanced green fluorescent protein (eGFP), pEGFP-N1 (Fig. 3A, group I). pEGFP-R6-VP1u, pEGFP-R7-VP1u, and pEGFP-R8-VP1u were generated by inserting the 5′ UTR of the R6, R7, and R8 transcripts, respectively, into pEGFP-VP1u (Fig. 3A, group II). The group III plasmids were constructed by deleting the 5′ UTR sequences in pEGFP-R6. pEGFP-T1, pEGFP-T2, and pEGFP-T3 were constructed by deleting exon 4, exons 3 and 4, and exons 3 and 4 plus part of exon 2 (nt 2174 to 2260), respectively. pEGFP-T4, pEGFP-T5, and pEGFP-T6 were constructed by deleting exon 1 plus part of exon 2 (nt 2140 to 2173), exons 1 and 2, and exons 1 and 2 plus part of exon 3 (nt 2332 to 2425), respectively. pEGFP-E3 and pEGFP-E4 harbored exon 3 and 4, respectively, of the R6-5′ UTR plus the VP1u upstream eGFP ORF. The plasmids in group IV were constructed by deleting exon 4 in plasmids pEGFP-R6-VP1u, pEGFP-R7-VP1u, and pEGFP-R8-VP1u.

### (iv) 5′ UTR uATG mutant plasmids.

p3XFlag-muATG1 was made by mutating nt 2213 from T to C in the p3XFlag-R6 plasmid. p3XFlag-muATG2, p3XFlag-muATG3, and p3XFlag-muATG2 and -3 were constructed by producing T3122C, T3143C, or both mutations, respectively, as indicated in Fig. 7A.

### (v) IRES reporter plasmids.

Bicistronic fluorescent reporter plasmids were constructed based on the pIRES2-eGFP vector (Clontech), as indicated in Fig. 5A. The mCherry gene with an N-terminal 3×Flag tag was inserted into the pIRES2-eGFP vector. The IRES2 sequence was replaced by multiple-cloning sites (MCS), and the plasmid was named pmCherry-EGFP. The encephalomyocarditis virus (ECMV) IRES was inserted as a positive control (pmCherry-IRES2-EGFP). pBi-R6, pBi-R7, pBi-R8, and pBi-VP1u were made by inserting the 5′ UTR of the R6, R7, and R8 transcripts or the VP1u sequence, respectively, into the MCS. The 5′ UTR sequences of the R6, R7, and R8 transcripts were inserted into pBi-VP1u to generate plasmids pBi-R6-VP1u, pBi-R7-VP1u, and pBi-R8-VP1u, respectively.

### (vi) Leaky scanning-related plasmids.

The sequences before the ATG of VP1 were mutated by inserting a Kozak sequence (GCCGCCACC) or an anti-Kozak sequence (ATATATTTT) into plasmid p3XFlag-R6 or pT7-R6 to generate p3XFlag-antikozak, p3XFlag-kozak, pT7-antikozak, and pT7-kozak (57). The p3XFlag-TAshift and pT7-TAshift plasmids were constructed by producing C3375A and A3376T mutations in p3XFlag-R6 and pT7-R6, respectively. The ATG of VP1 was mutated (T3153C) in plasmids p3XFlag-mVP1 and pT7-mVP1 to produce plasmids p3XFlag-mVP1 and pT7-mVP1, respectively.

### (vii) Mutated infectious clone plasmids.

pHBoV-muATG1 and pHBoV-muATG2 and -3 were constructed by producing a T2213C mutation or a T3122C plus a T3143C mutation, respectively.

### *In vitro* coupled transcription/translation assay.

VP protein *in vitro* coupled transcription/translation assays were carried out with a TNT T7 Quick coupled transcription/translation system (catalog no. L1170; Promega) according to the manufacturer's protocol. In brief, 1 μg template plasmid DNA, 0.5 μl PCR enhancer buffer, and 1 μl [^35^S]methionine (catalog no. NEG709A; PerkinElmer) were added to 20 μl master mix and incubated at 30°C for 90 min, followed by adding 20 μl 1× sodium dodecyl sulfate (SDS) gel-loading buffer and heating at 75°C for 5 min. Samples were loaded onto a 15% gradient SDS-polyacrylamide gel electrophoresis (PAGE) gel and run for 1.5 h. The gel was fixed and dried at 85°C for 1 h. The signals were detected with a Cyclone Plus system (PerkinElmer) and analyzed using OptiQuant software.

### Virus purification and viral genome DNA preparation.

HEK293T cells were cultured on 10-cm dishes and transfected with 10 μg plasmid per dish. The transfected cells were harvested at 48 h posttransfection, resuspended in 10 ml phosphate-buffered saline (PBS), and then lysed by four cycles of freezing and thawing. The lysates were treated with DNase I for 30 min at 37°C and centrifuged at 10,000 × *g* for 30 min at 4°C. The supernatant was collected, and the virus was harvested by cesium chloride (CsCl) gradient centrifugation. The viral genome was extracted using a TIANamp virus DNA/RNA kit (Tiangen) according to the manufacturer's standard protocol.

### RNA isolation.

The total RNAs from transfected cells were harvested using the TRIzol reagent (Ambion) according to the manufacturer's instructions.

### Real-time PCR and statistical analysis.

Real-time PCR was performed with SYBR green PCR master mix (Bio-Rad) on a CFX Connect real-time system (Bio-Rad). The primers used to detect NS1 were NS1-F (5′-TGCAGACAACGCCTAGTTGTTT-3′) and NS1-R (5′-CTGTCCCGCCCAAGATACA-3′). The pHBoV plasmid was used as a control to establish a standard curve. Means and standard deviations were calculated from the results of at least three independent experiments using GraphPad Prism statistical analysis software.

### Northern blotting.

Total RNAs (10 μg) prepared from plasmid-transfected cells were run on a 1.5% agarose gel containing 2.2 M formaldehyde for 12 h at 28 V, followed by transfer to a Hybond-N^+^ membrane and UV cross-linking. Probe detection was performed using a DIG luminescence detection kit II (Roche) according to the manufacturer's protocol. Signals were detected using a ChemiDoc MP imaging system (Bio-Rad).

### Southern blotting.

Isolation of low-molecular-weight DNA and Southern blotting were performed as previously described (27) with digoxigenin (DIG)-labeled DNA probes (Roche).

### RNase protection assay (RPA).

Ten micrograms total RNAs prepared from transfected cells was incubated with a ^32^P-labeled probe at 51°C overnight, followed by treatment with RNase A and RNase T1 for 1 h and then precipitation with 2 volumes of 100% ethanol for at least 30 min at −40°C. The RNAs were collected by centrifuging for 15 min at 4°C, and they were then separated by running on a 6% gradient PAGE gel for 2 h. The signals were detected with the Cyclone Plus system (PerkinElmer) and analyzed using OptiQuant software.

### Western blotting.

Cell lysates were prepared at 2 days posttransfection and separated by 10% or 15% gradient SDS-PAGE, followed by transfer onto a nitrocellulose membrane. Protein detection was carried out with anti-GFP antibody (catalog no. 66002; Proteintech), anti-Flag antibody (catalog no. F1804; Sigma), or antiactin antibody (catalog no. sc-47778; Santa Cruz Biotechnology) using standard protocols. Luminescent signals were detected using a ChemiDoc MP imaging system (Bio-Rad).

### Statistical analysis.

Statistical analysis of the results of the *in vitro* coupled transcription/translation assay, Western blotting, quantitative PCR (qPCR), and RPA was performed by using a two-tail unpaired *t* test in GraphPad Prism software. Data are presented as the means ± standard deviations (*n* = 3). All the experiments were repeated at least three times.
